# The General Election

**Published:** 1900-09-29

**Authors:** 


### The Hospital

A Journal of

The Medical Sciences and Hospital Administration.

Vol. XXVIII.—No. 731.] Edited by Sir Henry Burdett, K.C.B., and Solomon C. Smith, M.D., M.R.C.P. [September 29, 1900.

### The General Election.

The fifteenth Parliament of Queen Victoria, which will commence its work with the opening of the twentieth century, bids fair to be elected under conditions very favourable to its value and efficiency. The voice of the faddist is for the moment lost in the general acclaim of the nation; and the prominence of an overwhelming Imperial interest has relegated the screeching "anti" to his, or her, natural position of insignificance. Comparatively few candidates, perhaps even none, will be so much as tempted to bid for hustings popularity by pledges to confiscate the property of publicans, to hinder the progress of scientific inquiry, or to promote the diffusion of disease among the soldiers and sailors upon whom, as recent experience has reminded us, we may at any time be compelled to depend for the safety of the Empire. The war has brought home to every locality, and, it may almost be said, to every household, a conviction that, after all, the world is ruled by the strong hand, and that the advantages which have been gained, at the cost of so much blood and treasure, by the valour of our home and colonial forces, must not be dissipated by foolish and ineffective diplomacy, with its piteous strivings to please all parties at once. The shortcomings of some members of the Government have been only too conspicuous, but there is a profound conviction that the Government cannot now be changed otherwise than for the worse. The Little Englanders, bent upon inducing or compelling his country to take a back seat among the nations, the merely time-serving politician, who, as Bishop Earle would phrase it, puts his foot into disputable questions "tenderly, like a cat into water, and pulls it out again, and still something unanswered delays him;" all these, whatever or however numerous their varieties, cannot be trusted to deal firmly and wisely with a great crisis in history; and so, human nature being what it is, it is useless to lay too much stress upon obvious lack of foresight or absence of preparation, such as could hardly have been avoided by any but men of exceptional firmness, decision, and ability. It is unjust to blame politicians overmuch for defects which are really, in great measure, those of the system which they are called upon to administer; or to expect independent initiative from men whose habitual existence is a state of bondage to fools. Nothing but the rough lessons of the past year could have rendered the great bulk of the British electorate deaf to a repetition of the twaddle which was talked in extenuation of the South African surrender of 1881, a surrender the true motive of which has lately been admitted, by one of the politicians responsible for it, to have been the simple cowardice of men who, in the course of long lives devoted to the pursuit of vote catching, had failed to discover either the deep seated courage and determination of the English people, or the fact that their unstinted devotion and confidence are invariably bestowed upon leaders by whom courage and determination are conspicuously displayed. Three months of such statesmanship as that of Oliver Cromwell, or Pitt, or even Palmerston, exercised in 1880, would have saved England twenty years of humiliation, and a war of which we have still to count the cost. We cannot afford to incur any risk of that cost being thrown away.

The great questions of the government of our new colonies in South Africa, of the character of our future relations with China, and of the reform of the Army, will, without doubt, be the first which the coming Parliament will be called upon to consider; but, intercurrently with these, or in immediate succession to them, will be arrears of social legislation and of social problems, all eminently worthy of attention, and all likely to be more wisely decided, by reason of the comparative elimination of the faddist element, in the next House of Commons than in any of its recent predecessors. Some of these problems will have direct bearing upon the relations of medical science to the State; and a good augury with regard to them may be drawn from the recent action of Mr. Chamberlain in promoting research into the causes of intermittent fever, and of Mr. Long in his successful endeavour to banish hydrophobia from England. Mr. Long, especially, has shown a contempt for the outcries of ignorant selfishness which cannot be too highly extolled, and his removal to the Local Government Board would be cordially welcomed by members of the medical profession. The presence of plague at Glasgow compels us to admit that our defences against disease are less complete than could be desired; and the recent reports of the Board's inspectors, from places in which they have investigated epidemics of contagious disease, have shown the existence of a deplorable apathy, or a deplorable want of knowledge, among many local authorities, with regard to conditions of drainage and of water supply. The case of Southampton, with its rapidly-increasing trade and population, has lately been made the subject of public comment of the most unfavourable kind; and, unless the published reports have been greatly exaggerated, it presents the spectacle of a great seaport about as badly prepared as would be possible against contingencies which are more than likely to occur.

